# Coupled Gas-Exchange Model for *C*_4_ Leaves Comparing Stomatal Conductance Models

**DOI:** 10.3390/plants9101358

**Published:** 2020-10-14

**Authors:** Kyungdahm Yun, Dennis Timlin, Soo-Hyung Kim

**Affiliations:** 1 School of Environmental and Forest Sciences, College of the Environment, University of Washington, Seattle, WA 98195, USA; kdyun@uw.edu; 2 Adaptive Cropping Systems Laboratory, Agricultural Research Service, U.S. Department of Agriculture, Beltsville, MD 20705, USA; dennis.timlin@usda.gov

**Keywords:** gas-exchange, C_4_ photosynthesis, stomatal conductance, Ball–Berry, Medlyn

## Abstract

Plant simulation models are abstractions of plant physiological processes that are useful for investigating the responses of plants to changes in the environment. Because photosynthesis and transpiration are fundamental processes that drive plant growth and water relations, a leaf gas-exchange model that couples their interdependent relationship through stomatal control is a prerequisite for explanatory plant simulation models. Here, we present a coupled gas-exchange model for $C_{4}$ leaves incorporating two widely used stomatal conductance submodels: Ball–Berry and Medlyn models. The output variables of the model includes steady-state values of $CO_{2}$ assimilation rate, transpiration rate, stomatal conductance, leaf temperature, internal $CO_{2}$ concentrations, and other leaf gas-exchange attributes in response to light, temperature, $CO_{2}$, humidity, leaf nitrogen, and leaf water status. We test the model behavior and sensitivity, and discuss its applications and limitations. The model was implemented in Julia programming language using a novel modeling framework. Our testing and analyses indicate that the model behavior is reasonably sensitive and reliable in a wide range of environmental conditions. The behavior of the two model variants differing in stomatal conductance submodels deviated substantially from each other in low humidity conditions. The model was capable of replicating the behavior of transgenic $C_{4}$ leaves under moderate temperatures as found in the literature. The coupled model, however, underestimated stomatal conductance in very high temperatures. This is likely an inherent limitation of the coupling approaches using Ball–Berry type models in which photosynthesis and stomatal conductance are recursively linked as an input of the other.

## 1. Introduction

Leaf gas-exchange includes the processes of $CO_{2}$ assimilation and water vapor exchange by plant leaves. It is one of the most important processes for life on Earth as it provides carbohydrate for food and oxygen for respiration practically for all organisms. Because plant growth depends on photosynthesis, it is an essential building block of plant simulation models. Photosynthesis models range in complexity from correlative models based on radiation use efficiency where carbon assimilation is proportional to total irradiance absorbed by leaf surfaces, to models based on enzyme kinetics [1]. Integration of more mechanistic photosynthesis model has been a critical aspect in crop modeling to better understand and predict crop productivity under dynamic environments [2,3].

A coupled approach to modeling photosynthesis, stomatal conductance, and transpiration simultaneously for $C_{3}$ plants has been presented by a number of studies [4,5,6,7,8]. This approach usually combines the FvCB (Farquhar–von Caemmerer–Berry) $C_{3}$ photosynthesis model [9] with a model of stomatal conductance [5,10,11] and an energy balance equation. The coupled modeling approach can describe the photosynthetic behavior of leaves by taking into account the biochemical limitation for $CO_{2}$ assimilation (demand), as well as the stomatal and other biophysical limitations in $CO_{2}$ supply, linked to transpiration and leaf temperature. These models describe photosynthesis mechanistically based on its key biochemical and anatomical characteristics.

Similarly to the $C_{3}$ model, a simplified biochemical model is also available for $C_{4}$ photosynthesis that takes into account $CO_{2}$ concentrating mechanism with the anatomical and functional separation between the mesophyll and bundle sheath cells [12]. However, while a number of studies have adapted and applied the coupled modeling approach for $C_{3}$ leaves, its application in $C_{4}$ leaves has been limited, with few exceptions [13,14]. An open source implementation of coupled gas-exchange modeling in R language is available with an emphasis on $C_{3}$ leaves [15]. A photosynthesis model with an emphasis on plant hydraulic balance of crassulacean acid metabolism (CAM) plants is available in Python language [16]. Dynamic programming languages, such as Python and R, are often easier to use in an interactive session, but suffer from slow performance. Julia is a new dynamic programming language primarily designed for the use in scientific computing with performance in mind [17].

Leaf gas-exchange processes can be limited by internal and external stress factors, such as leaf nitrogen and soil water availability [18]. A process-based model should incorporate these stress responses for realistic representation of the leaf gas-exchange processes. Correlations between key enzymatic parameters and leaf nitrogen content were often derived to describe down-regulation of photosynthesis under non-optimal nitrogen availability [19,20,21]. Stomatal conductance submodel was extended to acknowledge soil water status via leaf water potential and control the amount of transpiration and associated photosynthetic activity [20,22,23].

Meanwhile, many existing coupled photosynthesis models have relied on an empirical relationship between photosynthesis and stomatal behavior as established by Ball, Woodrow, and Berry, often referred to as Ball–Berry model [10]. Medlyn model extended Ball–Berry model in a similar structure to provide a better theoretical interpretation [11]. Comparisons between the two models generally reported similar performance when compared under usual conditions [24,25].

In this study, we developed a coupled gas-exchange model for $C_{4}$ leaves to compare performance of two stomatal conductance models: Ball–Berry (BB) and Medlyn (MED). Simulation using the two variants of coupled model were carried out under multiple environmental conditions with a varying degree of humidity, $CO_{2}$ concentration, air temperature, and irradiance. Responses to the simultaneous application of nitrogen and water stress were also investigated. Only a small number of model parameters were calibrated to an observation dataset, while most parameter values were from existing literature and models. An application of the model to replicate a known experiment with transgenic plants under a range of temperature is also discussed.

## 2. Results

### 2.1. Gas-Exchange Model Calibration

**Figure 1:**
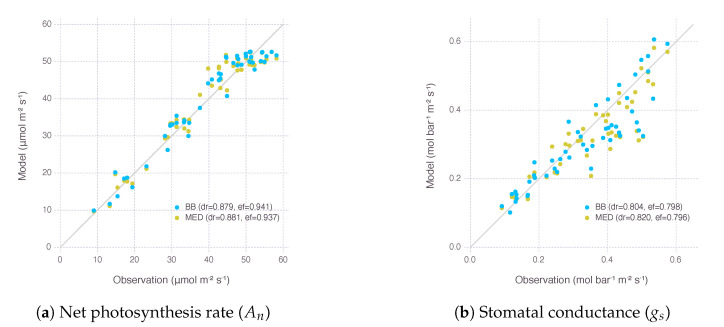
Evaluation of the gas-exchange model with two stomatal conductance submodels, Ball–Berry (BB) and Medlyn (MED), after calibrating parameters related to nitrogen ($N_{0}$, *s*) and stomata ($g_{0}$, $g_{1}$). Each dot represents an observed photosynthesis rate ($A_{n}$) or stomatal conductance ($g_{s}$) under a given experimental condition and a corresponding estimation by the model. A gray solid line shows 1:1 reference for comparison. dr indicates Willmott’s refined index of agreement ($d_{r}$), and ef indicates Nash–Sutcliffe modeling efficiency coefficient (NSE).

The performance of calibrated parameters was evaluated by Willmott’s refined index of agreement ($d_{r}$) and Nash–Sutcliffe model efficiency coefficient (NSE) for the two variants of gas-exchange model (Figure 1). $d_{r}$ of the model with Ball–Berry stomatal conductance submodel (BB) was 0.879 for net photosynthesis rate ($A_{n}$) and 0.804 for stomatal conductance ($g_{s}$). $d_{r}$ of the other model with Medlyn submodel (MED) was 0.881 for net photosynthesis rate ($A_{n}$) and 0.820 for stomatal conductance ($g_{s}$). NSE of the BB model was 0.941 for $A_{n}$ and 0.798 for $g_{s}$. NSE of the MED model was 0.937 for $A_{n}$ and 0.796 for $g_{s}$.

$d_{r}$ values were close to 1, meaning our models were calibrated adequately for further analysis carried in the following sections, especially when it was achieved by calibrating only four parameters, while most other parameters came from existing models or literature (Table A2). The two variants of the gas-exchange model with different selection of the stomatal conductance submodel did not show a clear difference in their performance within the range of input given by our experimental dataset.

**Figure A7:**
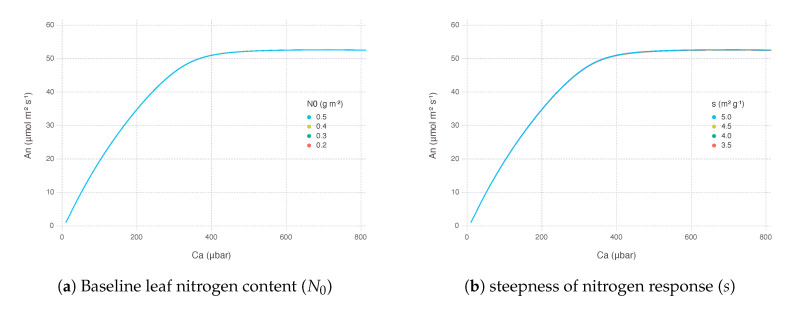
Sensitivity of parameters related to nitrogen dependence. The coupled gas-exchange model with Medlyn stomatal conductance model was used for testing. Air temperature ($T_{a}$) was 32 ${}^{\circ }C$, irradiance (*I*) was 2000 μmol_quanta_m^−2^s^−1^, and relative humidity (RH) was 66%.

Although the difference between two gas-exchange models only came from how stomatal conductance was calculated in submodel, the pooled nature of our calibration process produced a slightly different set of parameters used by the other parts of model, such as nitrogen submodel shared between the two variants. For example, with baseline leaf nitrogen content ($N_{0}$) and steepness of nitrogen response curve (*s*) parameters, BB got 0.371 and 4.470, while MED got 0.315 and 3.912, respectively. To ensure their difference did not hamper comparison between two stomatal conductance models, we did a further sensitivity analysis on the two other parameters in Appendix C and confirmed the difference was negligible in regards with $A_{n}$ response (Figure A7).

### 2.2. Stomatal Conductance Model Comparison

**Figure 2:**
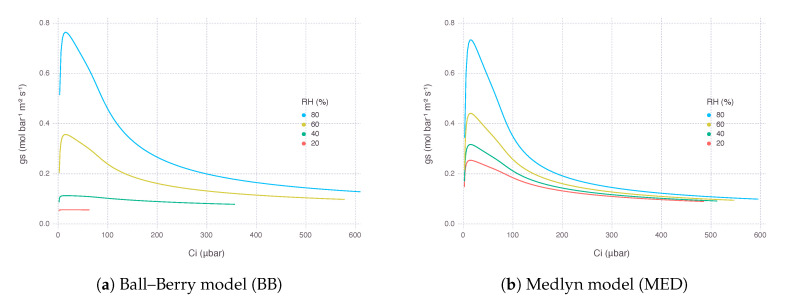
Stomatal conductance ($g_{s}$) estimated by two stomatal conductance models over a range of atmospheric $CO_{2}$ concentration ($C_{a}$) from 10 μbar to 1500 μbar at multiple levels of relative humidity (RH). The graph was plotted against resultant intercellular $CO_{2}$ concentration ($C_{i}$). Air temperature ($T_{a}$) was 32 ${}^{\circ }C$ and irradiance (*I*) was 2000 μmol_quanta_ m^−2^s^−1^.

$g_{s}$ predicted by MED was generally higher than $g_{s}$ from BB (Figure 2). $g_{s}$ from both models were in a similar range when relative humidity (RH) was around 65% to 90%, while maximum $g_{s}$ was much higher in MED when RH was saturated. When RH went lower than 50, gs from BB dropped rapidly and mostly converged to the lower bound ($g_{0_{\mathrm{BB}}}$), almost shutting down transpiration. In MED, decrease of $g_{s}$ along RH gradient was more gradual, and its value usually remained higher than the lower bound ($g_{0_{\mathrm{MED}}}$) to ensure a certain amount of transpiration keeps occurring.

**Figure 3:**
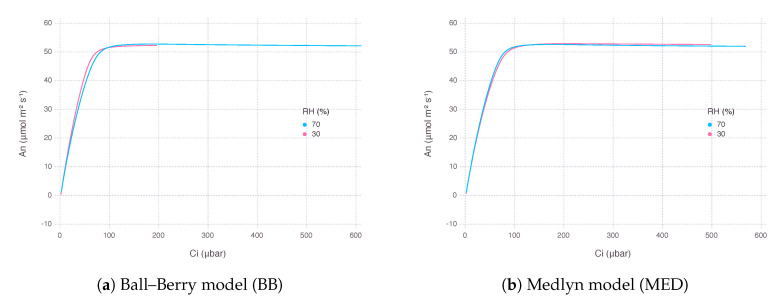
Net photosynthesis rate ($A_{n}$) estimated by two stomatal conductance models over a range of atmospheric $CO_{2}$ concentration ($C_{a}$) from 10 μbar to 1500 μbar at two levels of relative humidity (RH). The graph was plotted against resultant intercellular $CO_{2}$ concentration ($C_{i}$). Air temperature ($T_{a}$) was 32 ${}^{\circ }C$ and irradiance (*I*) was 2000 μmol_quanta_ m^−2^s^−1^.

As a result, $A_{n}$ from the gas-exchange model using BB decreased more rapidly, while the counterpart using MED showed a much gentle response as RH went down at the same level of concentration of atmospheric $CO_{2}$ ($C_{a}$). The rate of decrease in $A_{n}$ was 75% with BB and 20% with MED when RH was dropped from 80% to 20%. However, there was little difference in the curvature of $A_{n}$ response between the two models in terms of intercellular $CO_{2}$ concentration ($C_{i}$), suggesting the difference was mostly due to a change in supply function of the curve (Figure 3).

**Figure 4:**
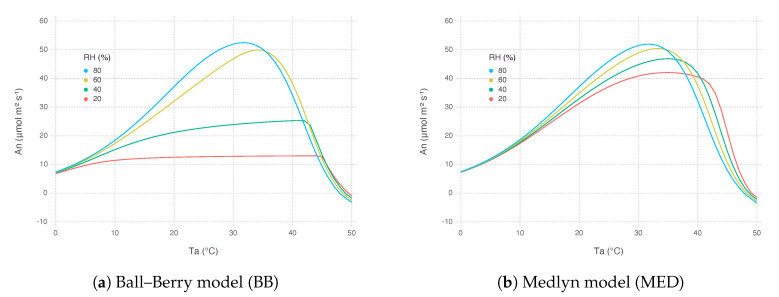
Net photosynthesis rate ($A_{n}$) estimated by two stomatal conductance models over a range of air temperature ($T_{a}$) from 0${}^{\circ }C$ to 50 ${}^{\circ }C$ at multiple levels of relative humidity (RH). Atmospheric $CO_{2}$ concentration ($C_{a}$) was 400 μbar and irradiance (*I*) was 2000 μmol_quanta_ m^−2^s^−1^.

A similar response can be observed when $A_{n}$ was plotted against a range of air temperature ($T_{a}$) from 0 ${}^{\circ }C$ to 50 ${}^{\circ }C$ (Figure 4). At lower RH, MED was able to maintain $g_{s}$ to a certain level and therefore keep $A_{n}$ from collapsing. The shifting of optimal temperature towards a higher regime with lower RH came from increased cooling effect by higher water loss in drier condition.

In turn, leaf temperature ($T_{l}$), which is adjusted by energy balance equation involving latent heat flux mainly driven by leaf transpiration, showed a clear difference between the two models. With BB, lower RH had stomata almost closed down and thus not able to cool down leaf temperature with latent cooling. On the contrary, MED implied higher latent cooling under lower RH due to stronger gradient of water vapor pressure formed between air and inside the leaf.

### 2.3. Stress Responses

#### 2.3.1. Leaf Nitrogen Deficiency

**Figure 5:**
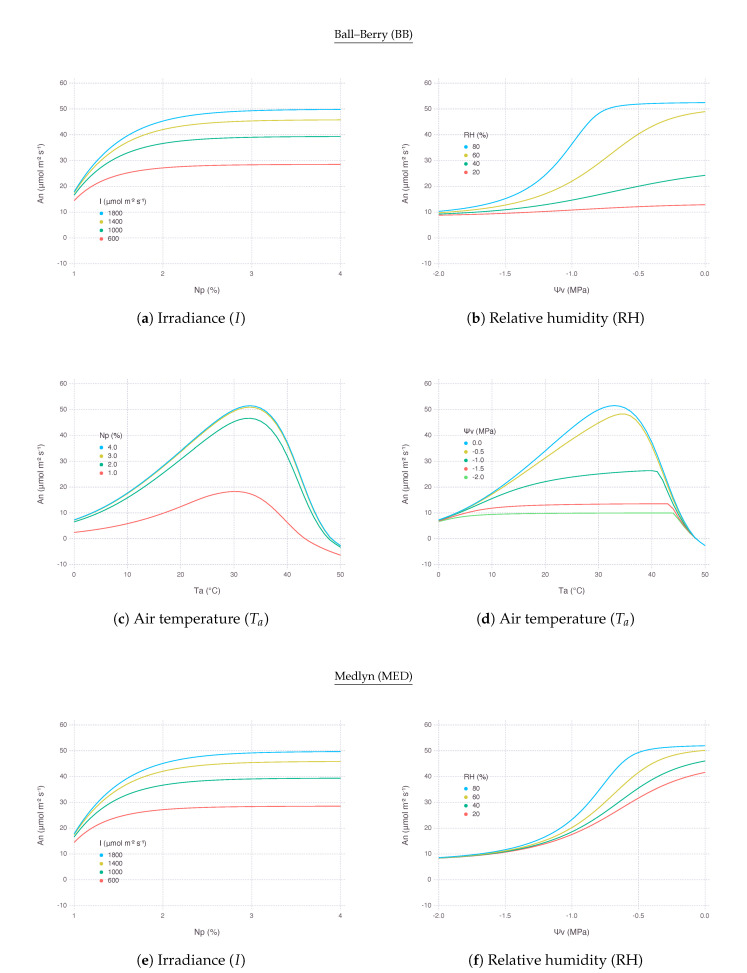

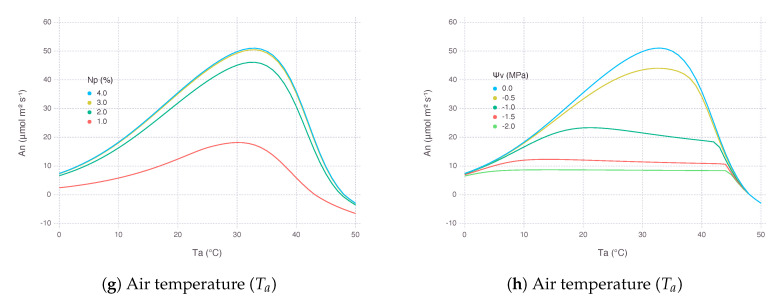
Net photosynthesis rate ($A_{n}$) over a range of environmental input variables at multiple levels of nitrogen or water stress for the two variants of coupled gas-exchange model using Ball–Berry (BB) and Medlyn (MED) stomatal conductance submodels. (**a**,**c**,**e**,**g**) Responses to a varying degree of relative leaf nitrogen content ($N_{p}$) assuming specific leaf area (SLA) was 200 cm^2^g^−1^. (**b**,**d**,**f**,**h**) Responses to bulk leaf water potential ($\Psi_{v}$).

$A_{n}$ generally decreased as leaf nitrogen content (*N*) reduced and the rate of decrease accelerated when nitrogen was more limited as represented by logarithmic curves. Yet, the strength of response was not constant and varied depending on environmental conditions. $A_{n}$ was more decreased with lower RH, but the difference diminished when *N* went below 0.5 g m^−2^ or relative leaf nitrogen content ($N_{p}$) was less than 1% assuming specific leaf area (SLA) was 200 cm^2^g^−1^. The rate of $A_{n}$ decrease did not change much with high atmospheric $CO_{2}$ ($C_{a}$) and only had more negative effect when $C_{a}$ was below 400 μmol mol^−1^. Response to $T_{a}$ under nitrogen stress was nonlinear that the decrease was more significant when $T_{a}$ was moving away from optimal temperature. The optimal temperature, where a peak $A_{n}$ could be achieved, slightly increased with more *N* available; thus, the maximum $A_{n}$ itself also increased due to more favorable biochemical reactions with higher temperature and *N* (Figure 5c,g). The slope of $A_{n}$ decrease by *N* stress was steeper under higher irradiance (*I*) and the difference between the levels of *I* gradually diminished as *N* approaching a minimum (Figure 5a,e). Overall, there was no clear difference in terms of nitrogen response between BB and MED models.

#### 2.3.2. Leaf Water Status

$A_{n}$ decreased as bulk leaf water potential ($\Psi_{v}$) reduced, but the rate of decrease did not monotonically change as in the case of nitrogen stress. Generally under a greater water stress with lower water potential, stress response represented by $A_{n}$ reduction tapered off and formed a logistic response curve. $A_{n}$ was more decreased with lower RH, but the difference diminished when $\Psi_{v}$ kept decreasing. In addition, note that with higher RH, leaf was able to sustain maximum $A_{n}$ even under mild water deficit which would have led to a noticeable reduction in $A_{n}$ under lower RH. For example, at −0.4 MPa, $A_{n}$ under 80% of RH did not decrease much, whereas $A_{n}$ under 40% of RH saw almost 60% reduction with BB (Figure 5b) and 20 reduction with MED (Figure 5f). The decrease of $A_{n}$ was more consistent with all range of $C_{a}$ compared to nitrogen stress response. For −1.5 MPa and below, the rate of $A_{n}$ decrease became almost identical regardless of $C_{a}$. With higher $C_{a}$, maximum $A_{n}$ was sustained for larger range of $\Psi_{v}$ similar to the RH response. Water response to $T_{a}$ was also nonlinear, but the difference wore off with lower $\Psi_{v}$ and no single optimal temperature was clear to be found (Figure 5d,h). Overall response to $T_{a}$ under water stress was similar between BB and MED, except BB exhibited much higher optimal temperature than MED in a mild-to-severe stress level, indicated by −1.0 MPa. Overall, BB was more sensitive to RH changes under water stressed conditions than MED (Figure 5d,f).

### 2.4. Interactions between Leaf Nitrogen and Water Potential

#### 2.4.1. Response to Relative Humidity

**Figure 6:**
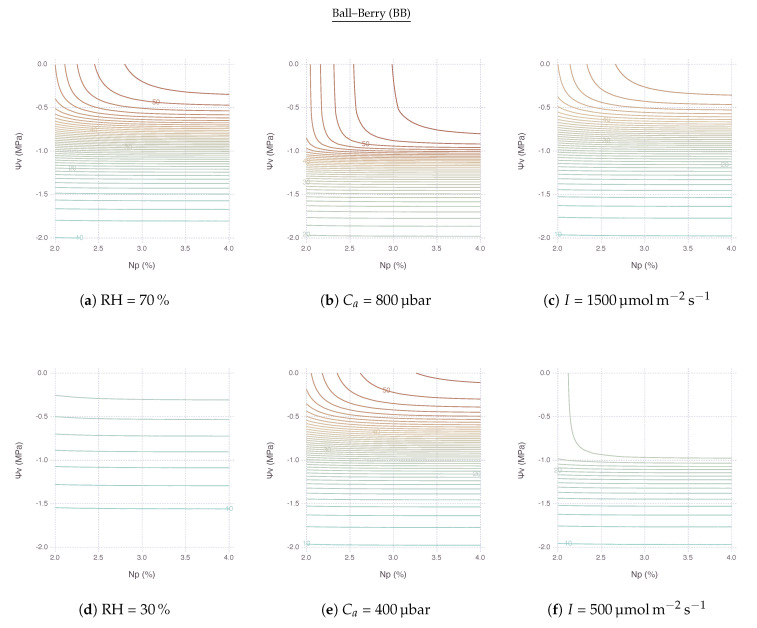

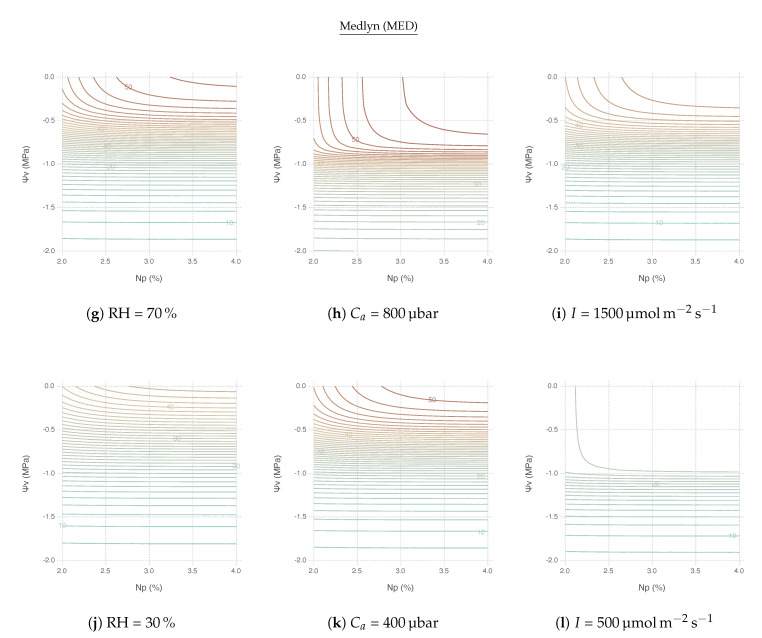
Responses of net photosynthesis rate ($A_{n}$) under nitrogen and water stress implied by relative leaf nitrogen content ($N_{p}$) and bulk leaf water potential ($\Psi_{v}$) for the two variants of coupled gas-exchange model using Ball–Berry (BB) and Medlyn (MED). (**a**,**d**,**g**,**j**) Contrasting effects of high and low relative humidity. (**b**,**e**,**h**,**k**) Comparison between elevated and current concentrations of atmospheric $CO_{2}$ ($C_{a}$). (**c**,**f**,**i**,**l**) Comparison between high and low levels of irradiance (*I*). Contour lines are spaced at an interval of 1 μmol m^−2^s^−1^ of the simulated $A_{n}$ values.

At high RH, $A_{n}$ remained relatively stable within its optimal range when both *N* and $\Psi_{v}$ kept high for less stress (Figure 6a,g). An area of this region located in the upper right side of contour plot shrunk and so did the range of non-limiting *N* and $\Psi_{v}$ as RH went down. In other words, with lower RH, $A_{n}$ became more sensitive to both nitrogen and water stress factors. The reduction of $A_{n}$ for lower RH was extremely strong with BB (Figure 6d) compared to MED (Figure 6j). No other variables showed such drastic difference between BB and MED in the comparison.

Note that, with low *N* and high $\Psi_{v}$, the sensitivity of $A_{n}$ was mostly governed by the change of *N*, thus limited by nitrogen. On the other hand, with high *N* and low $\Psi_{v}$, the most of sensitivity came from the change of $\Psi_{v}$, thus limited by water. Then, with both low *N* and $\Psi_{v}$, $A_{n}$ was also largely driven by $\Psi_{v}$ unless *N* dropped down to a very low range.

#### 2.4.2. Response to Atmospheric $CO_{2}$

The upper-right region of non-limiting $A_{n}$ existed with a range of $C_{a}$ above 400 μbar. This region vertically expands further down to cover lower $\Psi_{v}$ under higher $C_{a}$, as shown by comparing ambient $CO_{2}$ (Figure 6e,k) with elevated $CO_{2}$ (Figure 6b,h), indicating possible alleviation of water stress by elevated $CO_{2}$ concentration. $A_{n}$ remained relatively stable until $\Psi_{v}$ reached down to −1.0 MPa under 800 μbar of $C_{a}$, whereas $A_{n}$ started decreasing faster only after −0.5 Mpa under 400 μbar of $C_{a}$. Below these boundaries, $A_{n}$ was mostly limited by water only.

#### 2.4.3. Response to Irradiance

The region of non-limiting $A_{n}$ remained relatively stable with a range of *I* (Figure 6c,i) and even expanded further down when *I* was lower, although the magnitude of $A_{n}$ was much smaller (Figure 6f,l). In other words, under shaded condition with less light, nitrogen and water became less relevant because $A_{n}$ had to be much smaller. Below this region, $A_{n}$ was again mostly limited by water only unless *N* deficiency was extremely strong.

## 3. Discussion

### 3.1. Performance of Stomatal Conductance Models

We did not find much difference in performance between two stomatal conductance submodels BB and MED when used for fitting calibration dataset (Figure 1). It is known that two models have equal predictive strength for non-extreme environmental conditions where the data are usually collected for calibration and validation [25]. Results started deviating from each other under more extreme conditions, such as low $C_{a}$ and low RH, which are also close to the range where gas-exchange instruments often find difficulties in accurate measurements (Figure 2, Figure 3, Figure 4 and Figure 5b,f). Under low RH, especially below 50%, $g_{s_{\mathrm{BB}}}$ had much higher rate of decrease and almost converged to $g_{0_{\mathrm{BB}}}$ in the end, whereas $g_{s_{\mathrm{MED}}}$ maintained higher than $g_{0_{\mathrm{MED}}}$ with smooth and gentle transitions most of the time (Figure 2). Seemingly degenerate behavior of $g_{s_{\mathrm{BB}}}$ might be an overlooked effect of $g_{0_{\mathrm{BB}}}$ as a parameter estimated from regression rather than a directly measured value [26]. A clearly different response from each model warrants caution, especially when applying models to extreme conditions, such as concurrent exposure to high temperature and high vapor pressure deficit (VPD), likely encountered in future climate projections [27].

### 3.2. Stress Response to Elevated $CO_{2}$

Nitrogen stress consistently posed a negative impact on $A_{n}$ although the degree of reduction may vary depending on the severity of stress and corresponding environmental conditions (Figure 5a,c,e,g). While water stress also reduced $A_{n}$ in most of the time (Figure 5b,d,f,h), we observed in some conditions that the impact of water stress greatly diminished, for instance, when RH was higher than 60% (Figure 6a,g) or $C_{a}$ was higher than 400 μmol mol^−1^ (Figure 6b,h). There is increasing evidence that elevated $CO_{2}$ concentration alleviates water stress [28,29,30,31,32,33,34]. Our simulation result confirms this positive effect of elevated $CO_{2}$ by showing high $A_{n}$ sustained in a wider range of $\Psi_{v}$ under high $C_{a}$ due to reduced $g_{s}$ preserving water loss via transpiration. The effect was mostly pronounced under a mild water stressed condition since maximum $A_{n}$ did not change much in the absence of water stress [35]. Alleviation strength was dependent on leaf nitrogen supply such that even very mild water stress could not be overcome by high $C_{a}$ under low *N* [33].

### 3.3. Interactions between Nitrogen Deficiency and Water Stress

When both nitrogen and water stress were imposed simultaneously, the net effect of stress may vary depending on the relative degree of each stress factor and environmental variables. In the contour plots of interactive stress effects, we can identity three types of contours (Figure 5). Mostly vertical lines with very steep slopes indicate $A_{n}$ could be only improved by moving across a horizontal gradient, conferring a dominant *N* sensitivity. Likewise, mostly horizontal lines with a slope close to zero indicate $A_{n}$ could be only improved by moving across a vertical gradient, conferring a dominant $\Psi_{v}$ sensitivity. Other curves from round contour lines indicate $A_{n}$ are affected by both stress factors. With that in mind, looking back stress interaction result figures gives an insight that a vast area of *N* and $\Psi_{v}$ grid are covered by horizontal lines; thus, they are mostly under water limiting conditions. Nitrogen limiting conditions are, on the other hand, exhibited within an area where *N* is low or $\Psi_{v}$ is high. Such distinctions could be also found in literature where the negative impact of nitrogen stress could be inflated by an existence of drought stress [30,36,37,38].

### 3.4. Response of Transgenic $C_{4}$ Leaves to Temperature

**Figure 7:**
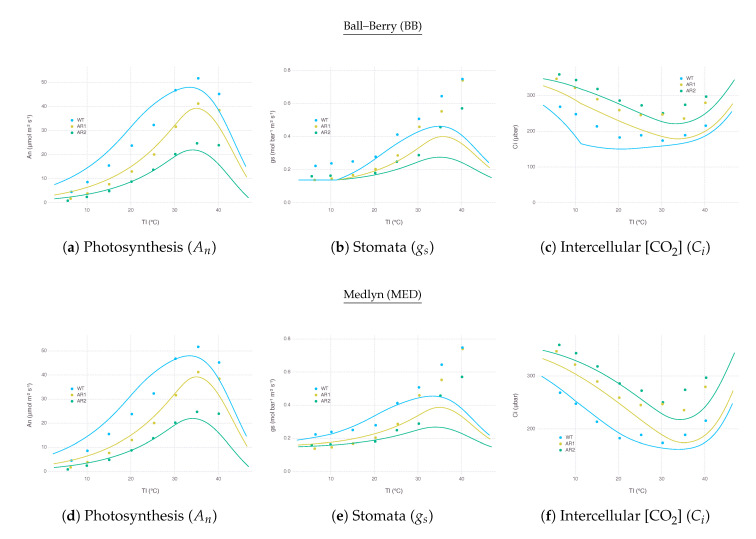
Gas-exchange simulation results over a range of leaf temperature for wild type (WT) and mutants (AR1, AR2) *Flaveria bidentis* leaves with Rubisco content reduced to 49% and 32%, respectively, to replicate Figure 1 of Kubien et al. [39]. Variables and parameters modified for this simulation are listed in Table A3. Other parameters remained the same as listed in Table A2. Solid curves represent model estimation for each treatment. Dots indicate data points digitized from the result of original experiment.

As an extended exercise for testing model response against real conditions, we applied our model to simulate a wide range of temperature response of transgenic $C_{4}$ plants, *Flaveria bidentis*, with varying amount of Rubisco reported by Kubien et al. [39]. By using a set of slightly modified parameters, as described in Appendix D, we were able to replicate overall patterns of $A_{n}$ (Figure 7d) and $C_{i}$ (Figure 7f) over a wide range of $T_{l}$ using MED. The performance of BB was close to MED, except that $C_{i}$, under low $T_{a}$ with more reduced Rubisco (AR2), showed an abrupt change due to $g_{s}$ that went too low, reaching the lower bound $g_{0_{\mathrm{BB}}}$ (Figure 7a,c).

Simulated $g_{s}$ closely followed the actual measurements until around 35 ${}^{\circ }C$ where model and observation started showing disagreement (Figure 7b,e). In the experiment, $g_{s}$ kept increasing further with higher $T_{l}$, whereas our model estimated $g_{s}$ should decrease when $A_{n}$ was not able to keep up under excessive heat stress. Such decreasing patterns occurred regardless of stomatal conductance submodels. This behavior is indeed contrary to observations where transpiration rate continually increases with higher leaf temperature, implying $A_{n}$ inhibition is not caused by stomatal closure; thus, $A_{n}$ should decouple from $g_{s}$ for some species [40,41]. However, this mechanism is not easily applicable to many if not all commonly used gas-exchange models relying on empirical relationship between $A_{n}$ and $g_{s}$, as explained in Appendix A.5. Alternative stomatal conductance models with mechanistic understandings on stomata responses to humidity and temperature could be useful for simulating field conditions where temperature fluctuates more than humidity [42,43]. In the meantime, more attention should be paid to the current results of gas-exchange models under high temperature, which is especially critical when simulating climate change scenarios.

## 4. Materials and Methods

### 4.1. Model Structure

The gas-exchange model consists of a number of smaller submodels describing different aspect of biochemical and physical processes coupled to each other following the structure of Kim and Lieth [7]. The $C_{4}$ photosynthesis is coupled with a stomatal conductance model via net photosynthesis rate ($A_{n}$) and also interacts with energy balance model to establish leaf temperature ($T_{l}$) [44]. We tested two stomatal conductance models, Ball–Berry model (BB) and Medlyn model (MED), combined with the rest of submodels remained the same, yielding two variations of the gas-exchange model. More details about the model specification are given in Appendix A.

### 4.2. Model Calibration

Most of parameter values came from existing literatures (Table A2). Some photosynthetic parameters were obtained from Pioneer hybrid 3733 maize (*Zea mays*) grown under Soil–Plant–Atmosphere Research (SPAR) chambers located at Beltsville, MD, USA, in 2002 [45]. Only four parameters were specifically calibrated for modeling experiments presented in this paper. Two are nitrogen-related parameters: baseline leaf nitrogen content ($N_{0}$) and steepness of nitrogen response curve (*s*). The other two are related to stomatal conductance: lower bound of stomatal conductance ($g_{0}$) and sensitivity of stomatal conductance ($g_{1}$). For comparison of overall gas-exchange response between two stomatal conductance models, calibration was separately done for the two models: Ball–Berry (BB) and Medlyn (MED) models.

$N_{0}$ and *s* calibrated for BB and MED were then pooled together to provide average parameter values used by the both models. To make sure a slight divergence in the parameter value did not make an impact on overall model response, we also conducted a sensitivity analysis on $N_{0}$ and *s* for a range of possible values. As $g_{0}$ and $g_{1}$ should involve inherent differences between two stomatal conductance models, a separate set of parameter values were used accordingly. BB model relied on $g_{0_{\mathrm{BB}}}$ and $g_{1_{\mathrm{BB}}}$. MED model relied on $g_{0_{\mathrm{MED}}}$ and $g_{1_{\mathrm{MED}}}$. The optimization method used for calibration was differential evolution algorithm [46].

The experimental dataset for calibration was collected from a growth chamber experiment conducted with Pioneer hybrid 34N43 maize in 2005 at Beltsville, MD, USA, as used in Kim et al. [44,47]. Spot measurements of gas-exchange were recorded with LI-COR LI-6400XT when plants were at the onset of reproductive stage. Three nitrogen levels at 0 kg ha^−1^, 50 kg ha^−1^ and 200 kg ha^−1^ in total were applied two times. For leaf nitrogen content (*N*), SPAD measurements were instead collected with Konica Minolta SPAD-502 and converted to *N*, as described in Appendix B.

The fitness of model calibration was evaluated by Willmott’s refined index of agreement $d_{r}$ as defined in Equation (1) [48] and Nash–Sutcliffe modeling efficiency coefficient (NSE) as defined in Equation (2) [49]. $y_{i}$ is an observed value for the variable of interest under a specific environmental condition ordered by *i*. ${\hat{y}}_{i}$ is an estimation by the model for the same input condition as $y_{i}$. $\bar{y}$ is the mean of observed values. Net photosynthetic rate ($A_{n}$) and stomatal conductance ($g_{s}$) were two variables selected for the evaluation.

(1) $$\begin{array}{cc} a & =\sum_{i}\left|{\hat{y}}_{i}-y_{i}\right|\\ b & =2\sum_{i}\left|y_{i}-\bar{y}\right|\\ d_{r} & =\left\{\begin{array}{cc} 1-\frac{a}{b} & \mathrm{if}\;a\leq b\\ \frac{b}{a}-1 & \mathrm{otherwise} \end{array}\right. \end{array}$$

(2) $$\begin{array}{cc} NSE & =1-\frac{\sum_{i}{\left({\hat{y}}_{i}-y_{i}\right)}^{2}}{\sum_{i}{\left(y_{i}-\bar{y}\right)}^{2}} \end{array}$$

### 4.3. Model Comparison

We compared two variants of the gas-exchange model depending on which submodel was used for calculating stomatal conductance: Ball–Berry (BB) and Medlyn (MED). Net photosynthetic rate ($A_{n}$) was calculated over a range of values for specific environmental input variables, atmospheric $CO_{2}$ and air temperature ($T_{a}$). $C_{a}$ ranged from 0 μbar to 1500 μbar ranged from −10 ${}^{\circ }C$ to 50 ${}^{\circ }C$. Other environmental variables remained constant as default values (Table 1). Since stomatal conductance can be more directly related to the relative humidity of surrounding air (RH) than other variables, a response curve of $A_{n}$ was separately obtained for ten levels of RH from 0% to 100%.

### 4.4. Model Response

We simulated nitrogen and water stress factors by adjusting relevant parameter values. For nitrogen stress, leaf nitrogen content (*N*) was changed from 0 g m^−2^ to 2 g m^−2^ to impose stress when *N* was lower. For water stress, bulk leaf water potential ($\Psi_{v}$) was changed from 0 MPa down to −3 MPa to impose stress when $\Psi_{v}$ was lower. The range of values were deliberately chosen by first selecting biophysically feasible extremes, 0 g m^−2^ for *N* and 0 MPa for $\Psi_{v}$, then selecting a value for the other end where output variable exhibits a clear convergence. When simulating a response for each stress factor, the other stress factor assumed to be not limiting. The response curve of net photosynthetic rate ($A_{n}$) was obtained for multiple environmental input variables including relative humidity (RH), atmospheric $CO_{2}$ ($C_{a}$), air temperature ($T_{a}$), and irradiance (*I*).

In order to observe interaction effects between the two stress factors, contour plot of $A_{n}$ was obtained by applying the same range of two stress factors simultaneously. For each environmental input variable, four different levels of the value were chosen to show a varying contrast as the input changes. RH changed to 30, 50, 70 and 90%. $C_{a}$ changed to 200, 400, 600 and 800 μbar. *I* changed to 500, 1000, 1500 and 2000 μmol m^−2^s^−1^.

## 5. Conclusions

We compared how two stomatal conductance models, Ball-Berry (BB) and Medlyn (MED), perform when coupled with the $C_{4}$ photosynthesis model of von Caemmerer [12]. Traditionally, BB has been widely used in leaf gas-exchange modeling research. More recently proposed MED approach is founded upon BB approach to provide physiological underpinnings of empirical parameters and has been considered as an alternative to BB. Our results confirmed that the performance of two models were comparable in a wide range of environmental conditions, yet could deviate substantially in low humidity conditions.

We further tested applicability of the model by replicating the behavior of transgenic $C_{4}$ leaves under moderate temperatures found in the literature. The coupled model, however, underestimated stomatal conductance in very high temperatures, presumably due to an inherent limitation of the coupling approaches using Ball–Berry type models in which photosynthesis and stomatal conductance are recursively linked as an input of the other.

We were able to reach these findings thanks to a novel modeling framework written in Julia programming language used for model development and analysis [50]. Our modeling approach is extensible and can be a useful means for studying the ecophysiology of $C_{4}$ plants, including staple and energy crops, such as maize, sorghum, and switchgrass.

## Figures and Tables

**Table 1:** Default values for environmental input variables.

Symbol	Value	Units	Description
$C_{a}$	400	μbar	Atmospheric $CO_{2}$ partial pressure
$P_{a}$	99.4	kPa	Atmospheric pressure
$T_{a}$	32	${}^{\circ }C$	Air temperature in Celsius
*I*	2000	μmol_quanta_ m^−2^s^−1^	Incident PAR
*u*	2	m s^−1^	Wind speed
RH	66	%	Relative humidity of the air
SPAD	60	-	SPAD value

**Figure A1:**
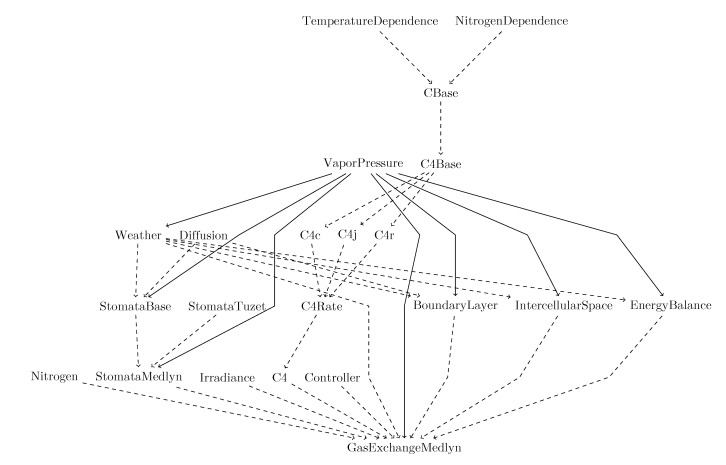
System diagram of the gas-exchange model (GasExchangeMedlyn) incorporating several submodels including Medlyn stomatal conductance model implemented on Cropbox framework. Solid arrow indicates ‘has’ relationship that the entity pointed out by the arrow owns the other entity and takes responsibility of state updates. Dashed arrow indicates ‘is’ relationship that the pointing entity becomes a trait of the other entity at the end.

**Figure A2:**
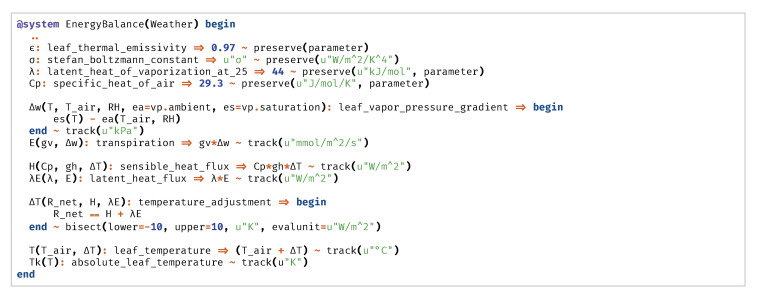
Snippet of model code implementing energy balance process.

**Figure A3:**
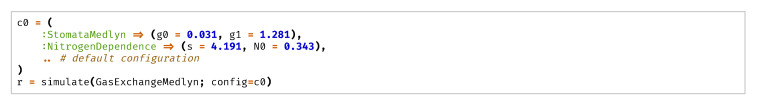
Script for running a coupled gas-exchange model with Medlyn stomatal conductance submodel. Parameter values as listed in Table A2 are included in the baseline configuration c0 used by simulate() function.

**Figure A4:**
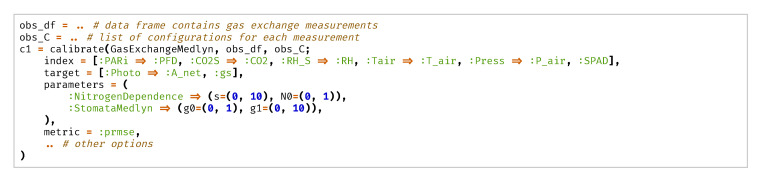
Script for calibrating parameters for the gas-exchange model. index option provides a mapping of variable names between a dataset and the model specification. target option indicates two variables, $A_{n}$ and $g_{s}$, for which fitting errors are minimized. parameters option specifies a range of parameter values to be calibrated. A set of calibrated parameters are stored in a variable named c1.

**Figure A5:**
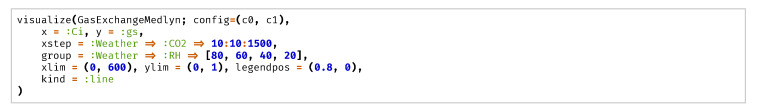
Script for generating Figure 2b. visualize() function internally runs simulate() and render a plot using the output. Note that a configuration used here is the baseline c0 partially overridden by the calibrated c1. x and y options specify variables used for two axes of the plot. xstep option provides a range of value to compose a single curve, indicating atmospheric $CO_{2}$ concentration ($C_{a}$) is ranged from 10 μbar to 1500 μbar. group option provides a group of treatments to render multiple curves, indicating four levels of relative humidity (RH) are used. xlim option sets the visible range of $C_{i}$ limited from 0 μbar to 600 μbar and ylim option sets the range of $g_{s}$ from 0 to 1 mol_H_2_O_m^−2^s^−1^bar^−1^. kind option for plot type can be either :line or :scatter.

**Figure A6:**
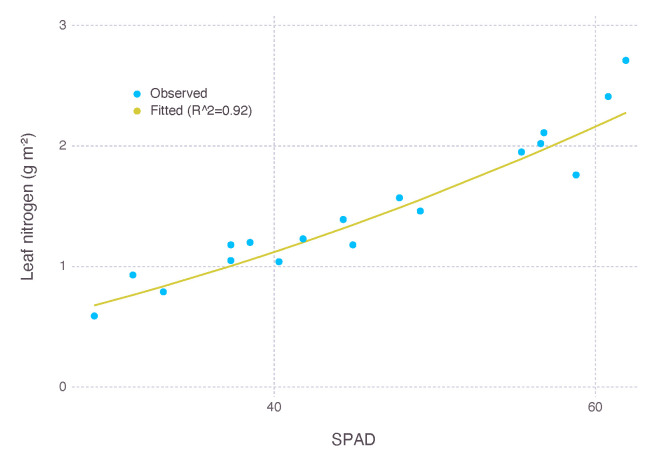
Fitting SPAD measurement to leaf nitrogen content (*N*). *N* was fitted by a quadratic equation $N=0.0004x^{2}+0.012x$ for 18 samples of SPAD measurement (x=SPAD) with $R^{2}=0.92$.

## Abbreviations

The following abbreviations are used in this manuscript:

BB	Ball–Berry stomatal conductance model [10]
CAM	Crassulacean Acid Metabolism
FvCB	Farquhar–von Caemmerer–Berry model [9]
MED	Medlyn stomatal conductance model [11]
NSE	Nash–Sutcliffe model efficiency coefficient [49]
PAR	Photosynthetic Active Radiation
PEPC	Phosphoenolpyruvate Carboxylase
PPFD	Photosynthetic Photon Flux Density
PRMSE	Percentage Root Mean Square Error
PSII	Photosystem II
RH	Relative Humidity
SLA	Specific Leaf Area
SPAD	Soil Plant Analysis Development
SPAR	Soil–Plant–Atmosphere Research
VPD	Vapor Pressure Deficit
WT	Wild type
AR1, AR2	Mutants with reduced Rubisco content

## Appendix A. Gas-Exchange Model Specification

### Appendix A.1. C_4_ Photosynthesis

The biochemical demand for $CO_{2}$ assimilation in $C_{4}$ leaves was adapted from an existing $C_{4}$ photosynthesis model [12]. The rate of net $CO_{2}$ assimilation ($A_{n}$) was represented by the minimum of enzyme limited ($A_{c}$) and electron transport limited ($A_{j}$) $CO_{2}$ assimilation rates with curvature factor β.

(A1) $$A_{n}=\min\;h\left\{A_{c},A_{j},\beta \right\}$$

The transition between $A_{c}$ and $A_{j}$ was calculated using a hyperbolic minimum where minh{a,b,c} is equivalent to taking the lower root of quadratic equation $cx^{2}-\left(a+b\right)x+ab=0$ with curvature factor *c* interpreted as a parameter of co-limitation [51,52]. $A_{c}$ can be approximated by the minimum of phosphoenolpyruvate carboxylase (PEPC) activity ($A_{c_{1}}$) and Rubisco activity ($A_{c_{2}}$) taking into account the bundle-sheath leakage and mitochondrial respiration. $A_{c}$ is driven by $A_{c1}$ under low [$CO_{2}$] and by $A_{c2}$ under high [$CO_{2}$].

(A2) $$\begin{array}{cc} A_{c} & =\min\{A_{c_{1}},A_{c_{2}}\} \end{array}$$

(A3) $$\begin{array}{cc} A_{c_{1}} & =V_{p}+g_{bs}C_{m}-R_{m} \end{array}$$

(A4) $$\begin{array}{cc} A_{c_{2}} & =V_{c\;\max}-R_{d} \end{array}$$

$V_{p}$ is the rate of $C_{4}$ carboxylation and assumed to be limited either by PEPC activity or PEP regeneration. $g_{bs}$ is the bundle sheath conductance to $CO_{2}$ and $C_{m}$ is the mesophyll $CO_{2}$ partial pressure. $V_{c\;\max}$ is the maximum rate of Rubisco carboxylation (μmol m^−2^s^−1^). $R_{d}$ is mitochondrial respiration in the light (μmol_CO_2__ m^−2^s^−1^) and half of its value was assumed a mesophyll component, $R_{m}$.

(A5) $$V_{p}=\min\left\{\frac{C_{m}V_{p\;\max}}{C_{m}+K_{p}},V_{pr}\right\}$$

$V_{p\;\max}$ is the maximum PEP carboxylation rate, $K_{p}$ is the Michaelis-Menton constant for $CO_{2}$ of PEPC, and $V_{pr}$ is PEP regeneration rate. Provided that the resistance for $CO_{2}$ from intercellular spaces to mesophyll cells is negligible, $C_{m}$ can be estimated from $CO_{2}$ partial pressure of the air ($C_{a}$) after taking account of total leaf resistance to $CO_{2}$ ($r_{v_{c}}$) under a given $A_{n}$.

(A6) $$\begin{array}{cc} C_{m} & \approx C_{i}=C_{a}-A_{n}r_{v_{c}} \end{array}$$

(A7) $$\begin{array}{cc} r_{v_{c}} & =r_{s_{c}}+r_{b_{c}} \end{array}$$

(A8) $$\begin{array}{cc} r_{s_{c}} & =\frac{1}{g_{s}}\;\cdot \;{\left(\frac{D_{w}}{D_{c}}\right)}^{\;1} \end{array}$$

(A9) $$\begin{array}{cc} r_{b_{c}} & =\frac{1}{g_{b}}\;\cdot \;{\left(\frac{D_{w}}{D_{c}}\right)}^{\;\frac{2}{3}} \end{array}$$

The ratio between diffusion coefficient for water vapor ($D_{w}$) and $CO_{2}$ ($D_{c}$) is used for converting from stomatal conductance ($g_{s}$) and boundary layer conductance ($g_{b}$) in terms of $CO_{2}$ into resistance in terms of water vapor ($r_{s_{c}}$, $r_{b_{c}}$). We assume $g_{s}$ is subject to still air and thus raised to the power of 1, whereas $g_{b}$ is subject to convective air with laminar flow and thus raised to the power of $\frac{2}{3}$ [53,54].

$C_{m}$ was solved numerically by using bisection method because its dependence on $A_{n}$ which is required in the calculation of $g_{s}$ forms a cyclic dependency difficult to be solved analytically.

The assimilation rate limited by electron transport ($A_{j}$) can be approximated similarly to $A_{c}$ by the minimum of electron transport limited rates in $C_{4}$ and $C_{3}$ cycles.

(A10) $$\begin{array}{cc} A_{j} & =\min\{A_{j_{1}},A_{j_{2}}\} \end{array}$$

(A11) $$\begin{array}{cc} A_{j_{1}} & =\frac{xJ}{2}-R_{m}+g_{bs}C_{m} \end{array}$$

(A12) $$\begin{array}{cc} A_{j_{2}} & =\frac{(1-x)J}{3}-R_{d} \end{array}$$

Total rate of electron transport (*J*) was modeled using a non-rectangular hyperbola which can be described with hyperbolic minimum (minh). *x* is a partitioning factor of *J*.

(A13) $$J=\min\;h\{I_{2},J_{\max},\theta \}$$

θ is curvature of response of electron transport to photosynthetically active radiation (PAR) and $J_{\max}$ is the maximum rate of electron transport. $I_{2}$ is effective radiation absorbed by Photosystem II (PSII) [7,55].

(A14) $$\begin{array}{cc} I_{2} & =\frac{\left(1-f\right)}{2}I_{a} \end{array}$$

(A15) $$\begin{array}{cc} I_{a} & =\alpha I \end{array}$$

*I* is the incident light in photosynthetic photon flux density (PPFD) and $I_{a}$ is PPFD absorbed by the leaf. α is leaf absorptance in PAR and assumed 1−δ where δ is the proportion of the incident light scattered by the leaf surface. *f* is the spectral correction factor. PAR assumes the wave band of solar radiation from 400 nm to 700 nm.

The temperature dependence of $V_{p\;\max}$, $V_{c\;\max}$, and $R_{d}$ was approximated by the Arrhenius equation ($k_{T_{A}}$) normalized at a base temperature.

(A16) $$k_{T_{A}}\left[T_{k},E_{a}\right]=\exp\;\left[\frac{E_{a}\left(T_{k}-T_{b_{k}}\right)}{R\;\cdot \;T_{k}\;\cdot \;T_{b_{k}}}\right]$$

$E_{a}$ is the activation energy varies by process, $T_{l}$ is the leaf temperature and $T_{b}$ is the base temperature assumed 25 ${}^{\circ }C$. $T_{l_{k}}$ and $T_{b_{k}}$ are corresponding absolute temperatures in Kelvin (K). *R* is the universal gas constant. Each temperature dependent parameter has a value at 25 ${}^{\circ }C$.

The temperature dependence of $K_{p}$ and $V_{pr}$ was assumed to be $Q_{10}$ of 2.0.

(A17) $$k_{T_{Q}}\left[T\right]=Q_{10}^{\frac{T-T_{b}}{10}}$$

The temperature dependence of $J_{\max}$ was modeled using a peaked function [56].

(A18) $$k_{T_{P}}\left[T_{k},E_{a},H,S\right]=k_{T_{A}}\left[T_{k},E_{a}\right]\;\cdot \;\left(1+\exp\;\left[\frac{S\;\cdot \;T_{b_{k}}-H}{R\;\cdot \;T_{b_{k}}}\right]\right){\left(1+\exp\;\left[\frac{S\;\cdot \;T_{k}-H}{R\;\cdot \;T_{k}}\right]\right)}^{-1}$$

$E_{a}$ and *R* are as defined above. *H* is the curvature parameter determining the rate of decrease above the peak temperature and *S* is the entropy factor.

Nitrogen dependence of $V_{p\;\max}$, $V_{c\;\max}$, and $J_{\max}$ was modeled by a logistic function for the current leaf nitrogen content (*N*) [20]. $N_{0}$ is a baseline value assumed for the leaf nitrogen content and *s* is the steepness of nitrogen response curve.

(A19) $$k_{N}=\frac{2}{1+\exp\;\left[-s\;\cdot \;\max\left\{N_{0},N\right\}-N_{0}\right]}-1$$

Temperature dependence ($k_{T}$) and nitrogen dependence ($k_{N}$) are multiplicative limiting factors. For example, electron transport rate ($V_{p\;\max}$) is scaled from $V_{p\;\max_{25}}$ by calculating $V_{p\;\max}=V_{p\;\max_{25}}\;\cdot \;k_{T_{A}}\left[T_{l},E_{ap}\right]\;\cdot \;k_{N}$ with the current leaf temperature ($T_{l}$) and an activation energy for PEPC ($E_{ap}$).

### Appendix A.2. Boundary Layer Conductance

Boundary layer conductance to water vapor ($g_{b}$) was derived from convective heat conductance of the leaf surface ($g_{H}$). Corresponding Nusselt number (Nu) and Reynolds Number (Re) were derived by assuming forced convection of streamline flow on flat plates [57]. *u* is wind speed (m s^−1^) and *d* is characteristic dimension of the leaf determined by leaf width *W* (cm) [53]. $D_{m}$ is kinematic viscosity of the air and $D_{h}$ is thermal diffusivity of the air. $D_{m}$ and $D_{h}$ were assumed temperature independent at 20 ${}^{\circ }C$.

(A20) $$\begin{array}{cc} g_{H} & =\frac{D_{h}\;\cdot \;\mathrm{Nu}}{d} \end{array}$$

(A21) Nu=0.60Re

(A22) Re=u·dDm

(A23) $$\begin{array}{cc} d & =0.72w \end{array}$$

$g_{H}$ (m s^−1^) is then scaled and converted to $g_{h}$ (mmol m^−2^s^−1^) using the Gas Laws [57]. $T_{a_{k}}$ is the absolute temperature of air (K).

(A24) $$g_{h}=g_{H}\;\cdot \;\frac{P_{a}}{R\;\cdot \;T_{a_{k}}}$$

$g_{b}$ was then derived from $g_{h}$ using a diffusion ratio between water vapor and heat transfer under forced convection [53,54]. Additionally, $g_{b}$ was normalized to the current atmospheric pressure ($P_{a}$) to make its units explicitly expressed in vapor pressure gradient rather than in unitless mole fraction.

(A25) $$g_{b}=\frac{g_{h}}{P_{a}}\;\cdot \;{\left(\frac{D_{w}}{D_{h}}\right)}^{\;\frac{2}{3}}$$

### Appendix A.3. Stomatal Conductance

We used two approaches for modeling stomatal conductance ($g_{s}$). $g_{s_{\mathrm{BB}}}$ is stomatal conductance following Ball–Berry (BB) model [10] and $g_{s_{\mathrm{MED}}}$ follows Medlyn (MED) model [11].

(A26) $$g_{s_{\mathrm{BB}}}=g_{0_{\mathrm{BB}}}+g_{1_{\mathrm{BB}}}\frac{h_{s}A_{n}}{C_{s}}f_{\Psi_{v}}$$

$g_{0_{\mathrm{BB}}}$ (mol_H_2_O_ m^−2^s^−1^bar^−1^) is residual stomatal conductance to water vapor at the light compensation point and $g_{1_{\mathrm{BB}}}$ is the empirical coefficient for the sensitivity of $g_{s}$ to other variables. $h_{s}$ is relative humidity at the leaf surface (as a fraction), $A_{n}$ is net photosynthesis, and $P_{a}$ is the partial pressure of the air.

$f_{\Psi_{v}}$ is a weight factor that adjusts stomatal conductance to the bulk leaf water potential ($\Psi_{v}$) where $\Psi_{f}$ is a reference potential and $s_{f}$ is a sensitivity parameter [8].

(A27) $$f_{\Psi_{v}}=\frac{1+\exp\;\;\left[s_{f}\Psi_{f}\right]}{1+\exp\;\;\left[s_{f}\left(\Psi_{f}-\Psi_{v}\right)\right]}$$

$g_{s_{\mathrm{MED}}}$ has a slightly different form compared to $g_{s_{\mathrm{BB}}}$ that uses vapor pressure deficit at the leaf surface ($D_{s}$) instead of $h_{s}$. $g_{0_{\mathrm{MED}}}$ (mol_H_2_O_ m^−2^s^−1^bar^−1^) has the same meaning as $g_{0_{\mathrm{BB}}}$ setting a lower bound of the conductance value. $g_{1_{\mathrm{MED}}}$ is similarly a sensitivity parameter, but in different units ($\sqrt{\mathrm{kPa}}$) and scale.

(A28) $$g_{s_{\mathrm{MED}}}=g_{0_{\mathrm{MED}}}+\left(1+\frac{g_{1_{\mathrm{MED}}}}{\sqrt{D_{s}}}\right)\frac{A_{n}}{C_{s}}f_{\Psi_{v}}$$

In both models, $C_{s}$ is $CO_{2}$ partial pressure at the leaf surface after taking account of boundary layer resistance to $CO_{2}$ ($r_{b_{c}}$) under a given $A_{n}$.

(A29) $$C_{s}=C_{a}-A_{n}r_{b_{c}}$$

$h_{s}$ in Ball–Berry model was obtained by solving an equation connecting diffusion pathways of boundary layer and stomatal interface. $h_{a}$ is relative humidity in the air. Humidity inside the intercellular space was assumed fully saturated. The resultant Equation (A30) is quadratic since $g_{s_{\mathrm{BB}}}$ has $h_{s}$ in its component as defined in Equation (A26).

(A30) $$\left(h_{s}-h_{a}\right)g_{b}=\left(1-h_{s}\right)g_{s_{\mathrm{BB}}}$$

$D_{s}$ in Medlyn model was obtained in a similar way. Water vapor pressure at the leaf surface ($w_{s}$), in the air ($w_{a}$), and in the intercellular space ($w_{i}$) were used instead of relative humidity values. The resultant Equation (A31) is quadratic since $g_{s_{\mathrm{MED}}}$ has $D_{s}$ in its component as defined in Equation (A28). In an actual implementation, the equation was solved in terms of $\sqrt{D_{s}}$ and squared later to cope with a symbolic equation solver internally used by our framework.

(A31) $$\left(w_{s}-w_{a}\right)g_{b}=\left(w_{i}-w_{s}\right)g_{s_{\mathrm{MED}}}$$

### Appendix A.4. Energy Balance

Leaf temperature ($T_{l}$) can be different from air temperature ($T_{a}$) due to energy balancing on the leaf surface.

(A32) $$T_{l}=T_{a}+\Delta T$$

The temperature difference (ΔT) is obtained by numerically solving the energy budget equation.

(A33) $$R_{n}-H-\lambda E=0$$

The equation indicates that the net radiation absorbed by the leaf ($R_{n}$) should equal to the loss by sensible heat flux (*H*) and latent heat flux (λE), assuming no heat is stored in the leaf.

(A34) $$\begin{array}{cc} R_{n} & =R_{sw}+R_{lw} \end{array}$$

(A35) $$\begin{array}{cc} R_{sw} & =\alpha_{s}kI \end{array}$$

(A36) $$\begin{array}{cc} R_{lw} & =2\epsilon \sigma (T_{a_{k}}^{4}-T_{l_{k}}^{4}) \end{array}$$

The absorbed net radiation ($R_{n}$) is composed of shortwave component driven by solar radiation ($R_{sw}$) and longwave component via thermal radiation ($R_{lw}$). Note that we assumed gas-exchange measured inside a small chamber and the temperature of chamber wall was equivalent to the surrounding air. $\alpha_{s}$ is absorption coefficient of the leaf for solar radiation and ϵ is thermal emissivity of the leaf. σ is Stefan-Boltzmann constant.

(A37) $$H=C_{p}g_{h}\Delta T$$

For the sensible heat flux (*H*), $C_{p}$ is specific heat of air and $g_{h}$ is the convective heat conductance of the leaf surface.

(A38) $$\begin{array}{cc} E & =g_{v}\Delta w \end{array}$$

(A39) $$\begin{array}{cc} g_{v} & =\frac{1}{\frac{1}{g_{s}}+\frac{1}{g_{b}}} \end{array}$$

(A40) $$\begin{array}{cc} D & =e_{s}\left[T_{l}\right]-e_{a} \end{array}$$

For the latent heat flux (λE), λ is the latent heat of vaporization for water. *E* is the transpiration rate calculated with leaf conductance ($g_{v}$) and vapor pressure gradient between leaf surface and the air (Δw). $e_{a}$ is ambient vapor pressure of air. $e_{s}$ is saturated vapor pressure at a given temperature (*T*).

(A41) $$e_{s}\left[T\right]=0.611\;\cdot \;\exp\;\left[\frac{17.502\;\cdot \;T}{240.97+T}\right]$$

### Appendix A.5. Coupling Submodels

The net photosynthetic rate ($A_{n}$) depends on mesophyll [$CO_{2}$] ($C_{m}\approx C_{i}$) through Equations (A2) and (A10). $C_{i}$ depends on stomatal conductance ($g_{s}$) through Equation (A6). $g_{s}$ then again depends on $A_{n}$ through Equations (A26) and (A28), forming a cyclic dependency solved by an iterative numerical method. In the meantime, many parameters for $A_{n}$ depends on temperature that the temperature of biochemical reaction site ($T_{l}$) should play an important role. Vapor pressure of intercellular space (pi) used for Medlyn stomatal conductance model can also change with $T_{l}$. The latent heat component of Equation (A33) solved for $T_{l}$ is then driven by leaf conductance ($g_{v}$) which depends on $g_{s}$ through Equation (A38).

Therefore, three submodels for photosynthesis, stomatal conductance, and energy balance are interdependent. We used a nested iterative procedure using the bisection method to solve this relation numerically. For initial condition, each variable was given a sensible range of minimum and maximum values. $C_{i}$ was assumed to be at least 0 μbar and lower than two times of $C_{a}$. ΔT, the difference between leaf temperature ($T_{l}$) and air temperature ($T_{a}$), was assumed in the range of −10 K and 10 K.

### Appendix A.6. Implementation

An early version of the model was implemented in C++ and released as a sample of peer-reviewed publication of computer code [58]. Then, the model was translated, calibrated, and visualized with Cropbox framework [50] written in Julia programming language [17]. In this framework, a small component of the model is encapsulated into a structure called system and each system is a collection of variables described in a declarative form which closely resembles mathematical equations defined above and specifications of variables and parameters (Table A1 and Table A2). The gas-exchange model was composed of 23 systems including a submodel for stomatal conductance (Figure A1).

The framework provides a number of unique types of variable declaration that can help hiding complexity of implementation details. For example, $h_{s}$ in Equation (A30) is declared as a solve variable which is automatically expanded and solved in terms of symbolic algebra. ΔT in Equation (A32) in conjunction with Equation (A33) is declared as a bisect variable which would automatically generate necessary code to implement a nested iterative solver, as described in Appendix A.5. An optimal value of ΔT that satisfies $R_{n}=H+\lambda E$ as Equation (A33) would be found out by applying bisection method (Figure A2). Given Equation (A6) was also implemented as a bisect variable, the framework would analyze dependency between variables and generate proper code for nested structure.

Note that all equations described in this section assume implicit units conversion and scaling which is a feature provided by Cropbox framework. For example, in Equation (A23), leaf width *W* was declared in ‘cm’ then automatically scaled to match the units of final product in ‘m’ that an actual code generated for calculating characteristic dimension was $d=0.72\left(w\;\cdot 0.01m/\mathrm{cm}\right)$.

**Table A1:** Variables declared in the model.

Symbol	Units	Description
$C_{4}$ Photosynthesis		
$A_{c}$	μmol_CO_2__m^−2^s^−1^	Rubisco-limited $CO_{2}$ assimilation rate
$A_{j}$	μmol_CO_2__m^−2^s^−1^	Electron transport-limited $CO_{2}$ assimilation rate
$A_{n}$	μmol_CO_2__m^−2^s^−1^	Net photosynthesis rate
$C_{a}$	μbar	Atmospheric $CO_{2}$ partial pressure
$C_{i}$	μbar	Intercellular $CO_{2}$ partial pressure
$C_{m}$	μbar	Mesophyll $CO_{2}$ partial pressure
*J*	μmol_electrons_m^−2^s^−1^	Electron transport rate
$J_{\max}$	μmol_electrons_m^−2^s^−1^	Maximum rate of electron transport
$k_{N}$	-	Nitrogen dependence
$k_{T_{A}}$	-	Temperature dependence by Arrhenius equation
$k_{T_{Q}}$	-	Temperature dependence by $Q_{10}$ function
$k_{T_{P}}$	-	Temperature dependence by a peaked function
$K_{c}$	μbar	Michaelis-Menton constant of Rubisco for $CO_{2}$
$K_{p}$	μbar	Michaelis-Menton constant of PEPC for $CO_{2}$
*N*	g m^−2^	Leaf nitrogen content
$N_{p}$	%	Relative leaf nitrogen content assuming SLA = 200 cm_2_g_−1_
$P_{a}$	kPa	Atmospheric pressure
$R_{d}$	μmol_CO_2__m^−2^s^−1^	Mitochondrial respiration rate
$r_{v_{c}}$	m^2^s ${\mathrm{mol}}_{{\mathrm{CO}}_{2}}^{-1}$ bar	Total leaf resistance to $CO_{2}$
$T_{a}$	${}^{\circ }C$	Air temperature in Celsius
$T_{a_{k}}$	K	Air temperature in Kelvin
$T_{l}$	${}^{\circ }C$	Leaf temperature in Celsius
$T_{l_{k}}$	K	Leaf temperature in Kelvin
$V_{p}$	μmol_CO_2__m^−2^s^−1^	$C_{4}$ carboxylation rate
$V_{pr}$	μmol_CO_2__m^−2^s^−1^	PEP regeneration rate
$V_{c\;\max}$	μmol_CO_2__m^−2^s^−1^	Maximum rate of Rubisco carboxylation
$V_{p\;\max}$	μmol_CO_2__m^−2^s^−1^	Maximum rate of $C_{4}$ carboxylation
Boundary Layer		
*d*	m	Leaf characteristic dimension
$g_{b}$	mol_H_2_O_m^−2^s^−1^bar^−1^	Boundary layer conductance to water vapor
$g_{h}$	mmol m^−2^ s^−1^	Leaf convective heat conductance in molar flux
$g_{H}$	m s^−1^	Leaf convective heat conductance
$r_{b_{c}}$	m^2^ ${\mathrm{mol}}_{{\mathrm{CO}}_{2}}^{-1}$ bar	Boundary layer resistance to $CO_{2}$
*u*	m s^−1^	Wind speed
Irradiance		
*I*	μmol_quanta_m^−2^s^−1^	Incident PAR
$I_{a}$	μmol_quanta_m^−2^s^−1^	Absorbed PAR
$I_{2}$	μmol_quanta_m^−2^s^−1^	Effective PAR
Stomatal Conductance		
$D_{s}$	kPa	Vapor pressure deficit at the leaf surface
$f_{\Psi_{v}}$	-	Water stress factor
$g_{s}$	mol_H_2_O_ m^−2^s^−1^ bar^−1^	Stomatal conductance to water vapor
$g_{s_{\mathrm{BB}}}$	mol_H_2_O_ m^−2^s^−1^ bar^−1^	Stomatal conductance to water vapor from Ball–Berry model
$g_{s_{\mathrm{MED}}}$	mol_H_2_O_ m^−2^s^−1^ bar^−1^	Stomatal conductance to water vapor from Medlyn model
RH	%	Relative humidity of the air (0–100)
$h_{a}$	-	Relative humidity of the air (0–1)
$h_{s}$	-	Relative humidity at the leaf surface (0–1)
$w_{a}$	kPa	Water vapor pressure in the air
$w_{i}$	kPa	Water vapor pressure in the intercellular space
$w_{s}$	kPa	Water vapor pressure at the leaf surface
$\Psi_{v}$	MPa	Bulk leaf water potential
$r_{s_{c}}$	m^2^s ${\mathrm{mol}}_{{\mathrm{CO}}_{2}}^{-1}$ bar	Stomatal resistance to $CO_{2}$
Energy Balance		
Δw	kPa	Vapor pressure gradient between leaf surface and the air
$e_{a}$	kPa	Vapor pressure in the ambient air
$e_{s}$	kPa	Saturated vapor pressure
*E*	mol_H_2_O_ m^−2^s^−1^	Transpiration rate
$g_{v}$	mol_H_2_O_ m^−2^s^−1^ bar^−1^	Total leaf conductance to water vapor
*H*	W m^−2^	Sensible heat flux
λE	W m^−2^	Latent heat flux
$R_{n}$	W m^−2^	Net radiation absorbed
ΔT	K	Temperature difference between $T_{l}$ and $T_{a}$

Input variables to the model (Table 1).

### Appendix A.7. Workflow

A system implemented on Cropbox framework can be run by simulate() function supplied with an optional configuration. For instance, the coupled gas-exchange model with Medlyn stomatal conductance submodel (GasExchangeMedlyn) was run with a set of default parameters described in Table 1 and Table A2 (Figure A3). simulate() function returns a result of simulation in a tabular data frame which can be further analyzed by various tools including own visualization methods provided by the framework. By default, the result contains values of all numerical variables declared in the controller system.

After testing models with initial parameters, calibration of four parameters, $N_{0}$, *s*, $g_{0_{\mathrm{MED}}}$ and $g_{1_{\mathrm{MED}}}$, as explained in Section 4.2 can be done by calibrate() function with a training dataset (Figure A4). In this example, we had two target variables in the dataset, Photo and gs, and wanted to compare them with A_net ($A_{n}$) and gs ($g_{s}$) estimated by the model. A percentage root mean square error (PRMSE) calculated from the difference between each set of variables was minimized by updating four parameters. The range of each parameter value is specified in parameters option.

**Table A2:** Parameters and constants used in the model.

Symbol	Value	Units	Description
$C_{4}$ Photosynthesis			
β	0.99	-	Sharpness of transition between $A_{c}$ and $A_{j}$
$E_{ac}$	55.9 [59]	kJ mol^−1^	Activation energy for $V_{c\;\max}$
$E_{aj}$	32.8	kJ mol^−1^	Activation energy for $J_{\max}$
$E_{ap}$	75.1	kJ mol^−1^	Activation energy for $V_{p\;\max}$
$E_{ar}$	39.8	kJ mol^−1^	Activation energy for $R_{d}$
$g_{bs}$	0.003 [12]	mol_CO_2__m^−2^s^−1^bar^−1^	Bundle-sheath conductance to $CO_{2}$
$H_{j}$	220 [44]	kJ mol^−1^	Curvature parameter for $J_{\max}$
$J_{\max_{25}}$	300	μmol_electrons_m^−2^s^−1^	Maximum rate of electron transport at 25 ${}^{\circ }C$
$K_{c_{25}}$	650 [12]	μbar	Michaelis-Menton constant of Rubisco for $CO_{2}$ at 25 ${}^{\circ }C$
$K_{p_{25}}$	80 [12]	μbar	Michaelis-Menton constant of PEPC for $CO_{2}$ at 25 ${}^{\circ }C$
$N_{0}$	0.343	g m^−2^	Baseline leaf nitrogen content
*R*	8.314	JK^−1^mol^−1^	Universal gas constant
$R_{d_{25}}$	2 [44]	μmol_CO_2__m^−2^s^−1^	Mitochondrial respiration rate at 25 ${}^{\circ }C$
*s*	4.191	m^2^g^−1^	Steepness of nitrogen response curve
$S_{j}$	702.6	J mol^−1^K	Entropy factor for $J_{\max}$
θ	0.5	-	Sharpness of transition between light limitation and saturation
$T_{b}$	25	${}^{\circ }C$	Base temperature in Celsius
$T_{b_{k}}$	298.15	K	Base temperature in Kelvin
$V_{c\;\max_{25}}$	50	μmol_CO_2__m^−2^s^−1^	Maximum rate of Rubisco carboxylation at 25 ${}^{\circ }C$
$V_{p\;\max_{25}}$	70	μmol_CO_2__m^−2^s^−1^	Maximum rate of PEP carboxylation at 25 ${}^{\circ }C$
$V_{pr_{25}}$	80 [12]	μmol_CO_2__m^−2^s^−1^	PEP regeneration rate at 25 ${}^{\circ }C$
*x*	0.4 [12]	-	Partitioning factor of electron transport rate
Irradiance			
α	0.85 [54]	-	Leaf absorptance in PAR
δ	0.15 [55]	-	Leaf scattering factor
*f*	0.15 [55]	-	Leaf spectral correction factor
Boundary Layer			
$D_{w}$	24.2	mm^2^s^−1^	Diffusion coefficient for water vapor in air at 20 ${}^{\circ }C$
$D_{c}$	14.7	mm^2^s^−1^	Diffusion coefficient for $CO_{2}$ in air at 20 ${}^{\circ }C$
$D_{h}$	21.5	mm^2^s^−1^	Diffusion coefficient for heat (thermal diffusivity) in air at 20 ${}^{\circ }C$
$D_{m}$	15.1	mm^2^s^−1^	Diffusion coefficient for momentum (kinematic viscosity) in air at 20 ${}^{\circ }C$
*W*	10	cm	Leaf width
Stomatal Conductance			
$g_{0_{\mathrm{BB}}}$	0.036	mol_H_2_O_m^−2^s^−1^bar^−1^	Lower bound of $g_{s_{\mathrm{BB}}}$
$g_{1_{\mathrm{BB}}}$	2.792	-	Sensitivity of $g_{s_{\mathrm{BB}}}$
$g_{0_{\mathrm{MED}}}$	0.031	mol_H_2_O_m^−2^s^−1^bar^−1^	Lower bound of $g_{s_{\mathrm{MED}}}$
$g_{1_{\mathrm{MED}}}$	1.281	$\sqrt{\mathrm{kPa}}$	Sensitivity of $g_{s_{\mathrm{MED}}}$
$\Psi_{f}$	−2.0	Mpa	Reference water potential
$s_{f}$	2.3 [23]	Mpa^−1^	Sensitivity of water response
Energy Balance			
$\alpha_{s}$	0.79 [60]	-	Radiation absorption coefficient of the leaf
$C_{p}$	29.3	J mol^−1^K^−1^	Specific heat of air
ϵ	0.97 [53]	-	Leaf thermal emissivity
*k*	0.22	J μmol^−1^	Radiation conversion factor
λ	44	KJ mol^−1^	Latent heat of vaporization at 25 ${}^{\circ }C$
σ	5.670 × 10^−8^	W m^−2^K^−4^	Stefan-Boltzmann constant

Parameter values calibrated in this paper. Parameter values calibrated with a dataset from [45].

Plotting model response in multiple ranges of criteria would require aggregation of results from models running multiple times with slightly different configuration. visualize() function takes care of a few common plotting scenarios in a simple interface. All figures in this paper were directly generated from the framework. For example, Figure 2b was generated by running GasExchangeMedlyn model with four levels of RH (RH) from 20% to 80% and plotting gs ($g_{s}$) against Ci ($C_{i}$) in lines (Figure A5). Each line of response was composed by changing CO2 ($C_{a}$) in a range from 10 μbar to 1500 μbar at an interval of 10 μbar.

A Jupyter notebook containing source code of the model with calibration datasets and scripts for producing figures presented in this paper is available at https://github.com/cropbox/plants2020.

## Appendix B. Calibration of SPAD Measurements

Our experimental dataset used in calibration had SPAD measurements as a proxy to leaf nitrogen content. A small number of samples were separately collected from direct measurement of leaf nitrogen content and then used to derive a relationship between SPAD measurement and leaf nitrogen content. A quadratic equation in the form of $N=ax^{2}+bx+c$ was fitted for leaf nitrogen content (*N*) and SPAD measurement (x=SPAD) where a=0.0004, b=0.012, and c=0 with $R^{2}=0.92$ (Figure A6).

## Appendix C. Sensitivity of Nitrogen Parameters

The sensitivity of parameters related to nitrogen dependence was obtained by calculating net photosynthesis rate ($A_{n}$) with a range of values for the parameter in question, while keeping other parameters unchanged (Figure A7). For reference, $N_{0}$ was 0.371 and 0.315, and *s* was 4.470 and 3.912, for BB and MED, respectively. Baseline leaf nitrogen content ($N_{0}$) barely had an impact on $A_{n}$ with its range from 0.2 g m^−2^ to 0.5 g m^−2^. Sensitivity of nitrogen response curve (*s*) did not make a large difference either within the range of calibrated parameter values. For example, two sets of $N_{0}$ and *s* mentioned above resulted into less than 1 difference in $A_{n}$ at 400 μmol m^−2^s^−1^ of ambient $CO_{2}$ level. Therefore we assumed the difference between nitrogen parameter values we obtained from calibration for BB and MED were negligible and safe to pool them together for one unified set of parameters. The averaged out values for $N_{0}$ and *s* used in the rest of simulation was 0.343 and 4.191 (Table A2).

## Appendix D. Parameters for Transgenic Plants

A few parameters were derived to replicate a wide range of temperature response of transgenic plants with reduced amounts of Rubisco reported by Kubien et al. [39]. We mostly used the same values for variables and parameters identified in the paper, except $J_{\max_{25}}$, which was not mentioned but needed adjustments to replicate original curves (Table A3). Measured values of $A_{n}$, $g_{s}$, and $C_{i}$ under three treatments were digitized from their Figure 1. WT indicates wild type, while AR1 and AR2 refers to mutants treated with anti-Rubisco antibody to have Rubisco content reduced to 49% and 32%, respectively.

**Table A3:** Variables and parameters for replicating experiment by Kubien et al. [39].

Symbol	Value	Units	Description
Input variables			
$C_{a}$	370	μbar	Atmospheric $CO_{2}$ partial pressure
*D*	12	mbar	Vapor pressure deficit of the air (converted to RH)
*I*	1500	μmol_quanta_m^−2^s^−1^	Incident PAR
Shared parameters			
$g_{0_{\mathrm{BB}}}$	0.138	mol_H_2_O_m^−2^s^−1^bar^−1^	Lower bound of $g_{s_{\mathrm{BB}}}$
$g_{0_{\mathrm{MED}}}$	0.138	mol_H_2_O_m^−2^s^−1^bar^−1^	Lower bound of $g_{s_{\mathrm{MED}}}$
Parameters for WT			
$E_{ac}$	56.1	kJ mol^−1^	Activation energy for $V_{c\;\max}$
$E_{ap}$	71.6	kJ mol^−1^	Activation energy for $V_{p\;\max}$
$J_{\max_{25}}$	300	μmol_electrons_m^−2^s^−1^	Maximum rate of electron transport at 25 ${}^{\circ }C$
$V_{c\;\max_{25}}$	51.48	μmol_CO_2__m^−2^s^−1^	Maximum rate of Rubisco carboxylation at 25 ${}^{\circ }C$
$V_{p\;\max_{25}}$	159.9	μmol_CO_2__m^−2^s^−1^	Maximum rate of PEP carboxylation at 25 ${}^{\circ }C$
Parameters for AR1			
$E_{ac}$	57.0	kJ mol^−1^	Activation energy for $V_{c\;\max}$
$E_{ap}$	69.4	kJ mol^−1^	Activation energy for $V_{p\;\max}$
$J_{\max_{25}}$	240	μmol_electrons_m^−2^s^−1^	Maximum rate of electron transport at 25 ${}^{\circ }C$
$V_{c\;\max_{25}}$	24.7	μmol_CO_2__m^−2^s^−1^	Maximum rate of Rubisco carboxylation at 25 ${}^{\circ }C$
$V_{p\;\max_{25}}$	186.3	μmol_CO_2__m^−2^s^−1^	Maximum rate of PEP carboxylation at 25 ${}^{\circ }C$
Parameters for AR2			
$E_{ac}$	59.3	kJ mol^−1^	Activation energy for $V_{c\;\max}$
$E_{ap}$	74.0	kJ mol^−1^	Activation energy for $V_{p\;\max}$
$J_{\max_{25}}$	120	μmol_electrons_m^−2^s^−1^	Maximum rate of electron transport at 25 ${}^{\circ }C$
$V_{c\;\max_{25}}$	15.54	μmol_CO_2__m^−2^s^−1^	Maximum rate of Rubisco carboxylation at 25 ${}^{\circ }C$
$V_{p\;\max_{25}}$	146.9	μmol_CO_2__m^−2^s^−1^	Maximum rate of PEP carboxylation at 25 ${}^{\circ }C$

Minimum $g_{s}$ observed in the dataset. Scaled to the ratio of maximum $A_{n}$ for each treatment to make up missing information.
